# Effect of influenza vaccines against mismatched strains: a systematic review protocol

**DOI:** 10.1186/2046-4053-1-35

**Published:** 2012-07-30

**Authors:** Andrea C Tricco, Ayman Chit, David Hallett, Charlene Soobiah, Genevieve Meier, Maggie Chen, Mariam Tashkandi, Chris Bauch, Mark Loeb

**Affiliations:** 1 Li Ka Shing Knowledge Institute of St Michael’s Hospital, Toronto, Ontario, Canada; 2 GlaxoSmithKline, Mississauga, Ontario, Canada; 3 Faculty of Pharmacy, University of Toronto, Toronto, Ontario, Canada; 4 Institute of Medical Sciences, University of Toronto, Toronto, Ontario, Canada; 5 Applied Health Research Centre, St. Michael's Hospital, Toronto, Ontario, Canada; 6 North America Vaccines Division, GlaxoSmithKline, Philadelphia, PA, USA; 7 Department of Mathematics and Statistics, University of Guelph, Guelph, Ontario, Canada; 8 McMaster University, Rm. 3203, 1200 Main Street West, Hamilton, Ontario, Canada

**Keywords:** Antigenic variation, Cross protection, Influenza A virus, Influenza B virus, Protocol, Systematic review, Vaccines

## Abstract

**Background:**

Influenza vaccines are most effective when the antigens in the vaccine match those of circulating influenza strains. The extent to which the vaccine is protective when circulating strains differ from vaccine antigens, or are mismatched, is uncertain. We propose to systematically review the cross-protection offered by influenza vaccines against circulating influenza A or B viruses that are not antigenically well-matched to vaccine strains.

**Methods/Design:**

This is a protocol for a systematic review and meta-analysis. Placebo-controlled randomized clinical trials (RCTs) reporting laboratory-confirmed influenza among healthy participants vaccinated with antigens of influenza strains that differed from those circulating will be included. The primary outcome is the incidence of laboratory-confirmed influenza (polymerase chain reaction (PCR) or viral culture). The secondary outcome is the incidence of laboratory-confirmed influenza through antibody assay (a less sensitive test than PCR or viral culture) alone or combined with PCR, and/ or viral culture. The review will be limited to RCTs written in English.

We will search MEDLINE, EMBASE, the Cochrane Central Register of Controlled Trials, previous influenza reviews, and the reference lists of included studies to identify potentially relevant RCTs. Two independent reviewers will conduct all levels of screening, data abstraction, and quality appraisal (using the Cochrane risk of bias tool).

If appropriate, random effects meta-analysis of vaccine efficacy will be conducted in SAS (version 9.2) by calculating the relative risk. Vaccine efficacy will be calculated using the following formula: (1 - relative risk × 100). The results will be analyzed by type of vaccine (live attenuated, trivalent inactivated, or other). Subgroup analysis will include the effects of age (children, adults, older participants), and influenza A versus influenza B on the results. For influenza B we will also consider variable degrees of antigenic mismatch (lineage and drift mismatch).

**Discussion:**

Our results can be used by researchers and policy-makers to help predict the efficacy of influenza vaccines during mismatched influenza seasons. Furthermore, the review will be of interest to patients and clinicians to determine whether to get immunized or support immunization for a particular influenza season.

## Background

Influenza causes a substantial medical and economic burden to society. In the US alone, approximately 31 million outpatient visits each year and approximately 3.1 million hospitalized days are attributable to influenza [1]. Direct medical costs related to influenza averaged US$10.4 billion in 2003 and the estimated lost earnings as a result of illness and loss of life accounted for US$16.3 billion [1]. The loss of productivity and time away from work can potentially hinder economic growth. For example, one study found that influenza and influenza-like illnesses accounted for 17.7% (1,389 of 7,868 days) of employee absenteeism [2].

Influenza is often diagnosed using clinical symptoms or signs, such as fever, respiratory illness, and myalgia. However, the clinical diagnosis of influenza, which is dependent on the prevalence of circulating influenza in the community, can be limited [3]. Diagnostic tests available for influenza include viral culture, serology, polymerase chain reaction (PCR), immunofluorescence assays, and rapid antigen testing. The sensitivity and specificity of all influenza diagnostic tests will vary by laboratory, type of test used, and type of specimen tested. However, PCR has emerged as the most reliable test that can be used in clinical care and research.

The most effective way to prevent influenza is vaccination. Safe and effective vaccines have been available and used for more than 60 years. Currently, the two major types of vaccine classification are trivalent inactivated vaccines (TIV) and live attenuated influenza vaccines (LAIV). Although previous reviews have examined the efficacy of these vaccines [4-6], none have focused on vaccine efficacy when circulating strains do not match the vaccine composition. Furthermore, previous reviews have not provided estimates of matched and mismatched vaccine efficacy specifically for influenza A and influenza B, which have different biological characteristics.

In this proposal, we describe a strategy to systematically review the crossprotection offered by influenza vaccines, which can be defined as protection against circulating influenza A or B viruses that are not antigenically wellmatched to the vaccine strains. Specifically, our research question is ‘what is the crossprotection afforded by vaccination (using an LAIV, TIV, or other type of vaccine) against influenza A or B and their subtypes and lineages?’

## Methods

The Preferred Reporting Items for Systematic Reviews and Meta-analyses (PRISMA) Statement will be used to guide the reporting and conduct of this review [7]. The systematic review protocol was registered with the PROSPERO database (registration number CRD42012001926).

### Eligibility criteria

Healthy children, adults or older participants were chosen as our population of interest. Influenza vaccines may be ineffective in immune-compromised individuals and those with chronic health conditions; therefore we will focus on healthy individuals. All influenza vaccines will be included, and will be categorized as TIV, LAIV, and others (that is, non-TIV or non-LAIV).

The eligibility criteria will be limited to randomized clinical trials (RCTs) and quasi-RCTs comparing influenza vaccine(s) with placebo. To be included, the RCTs must report data on the primary or secondary outcomes. The primary outcome is the incidence of laboratory-confirmed influenza identified by PCR or viral culture [8]. We will not include studies using rapid influenza diagnostic tests, as their sensitivity is low (especially during flu season) and false positives are common during low activity seasons [8]. Furthermore, we will not include laboratory-confirmed influenza through antibody assay as part of the primary outcome, as this is a less sensitive test than PCR and viral culture [8]. The secondary outcomes are laboratory-confirmed influenza identified by 1) antibody assay or 2) influenza infection determined by antibody assay, PCR, and/or viral culture. Inclusion of studies will not be limited by publication status or year of dissemination, however, only RCTs written in English will be included. The draft eligibility form can be found in Additional file 1: Appendix 1.

### Study selection process

The results from the literature search will be uploaded to online proprietary systematic review software (SysRevTool) available through the Li Ka Shing Knowledge Institute of St. Michael’s Hospital. This software will be used for screening the citations from the electronic database, as well as all full-text articles identified through the search.

To ensure reliability, a training exercise will be conducted prior to commencing the screening process. Using the inclusion and exclusion criteria, a random sample of 50 citations from the literature search will be screened by all reviewers. Inter-rater agreement will be calculated using a kappa statistic and percent agreement [9]. If poor to moderate agreement is observed (that is, a kappa statistic less than 0.6 or percentage agreement less than 60%), the inclusion and exclusion criteria will be revised as necessary.

Using the final relevance form, two reviewers will screen the literature search results for inclusion, independently. They will then independently review the full text of potentially relevant articles and screen them to determine inclusion using the same inclusion and exclusion criteria. Conflicts will be resolved by discussion or the involvement of a third reviewer.

### Information sources and search

Literature search strategies will be developed using medical subject headings (MeSH) and text words related to influenza vaccination. We will search MEDLINE (OVID interface, 1948 onwards), EMBASE (OVID interface, 1980 onwards), and the Cochrane Central Register of Controlled Trials (Wiley interface, current issue). The electronic database search will be supplemented by searching for trial protocols through *meta*Register (http://www.controlled-trials.com/mrct/). The literature search will be limited to the English language and human subjects.

To ensure literature saturation, we will scan the reference lists of included studies or relevant reviews identified through the search. We will also search the authors’ personal files to make sure that all relevant material has been captured. Finally, we will circulate a bibliography of the included articles to the systematic review team, as well as to influenza experts identified by the team.

An experienced information specialist (Dr. Jessie McGowan) will conduct all of the literature searches. The search strategy will be peer reviewed by another librarian using the Peer Review of Electronic Search Strategies (PRESS) checklist [10]. The search strategy for the main electronic database search in MEDLINE is presented in Additional file 2: Appendix 2.

### Data items

The data abstracted will include study characteristics (for example, year of conduct, sample size, setting, country of conduct, circulating viral strain(s), vaccine composition, viral strain(s) tested, infection detection method (for example, PCR, viral culture, assay type, type of vaccines examined); participant characteristics (for example, population, mean age and standard deviation, percent gender); and number of influenza infections per treatment group.

### Data collection process

A draft data abstraction form will be developed, piloted, and modified as necessary. To ensure data accuracy, two reviewers will independently abstract all of the data using the standardized data abstraction form. Discrepancies will be resolved by discussion amongst the review team.

We suspect that in some instances, multiple study publications will report data from the same population (that is, companion reports). When this occurs, the report with the largest sample size or longest duration of follow-up will be included and will be used to abstract data. The other report(s) will be used for supplementary data only. We will contact the study author(s) for further information when the data are not clearly reported.

### Risk of bias appraisal

We will appraise the risk of bias in the RCTs using the Cochrane risk of bias tool [12]. This tool is composed of the following seven items: selection bias (random sequence generation and allocation concealment), performance bias (blinding of participants and personnel), detection bias (blinding of outcome assessment), attrition bias (incomplete outcome data), reporting bias (selective outcome reporting), and other sources of bias. For the selective outcome reporting criterion, the RCT protocols will be searched for using the *meta*Register database (http://www.controlled-trials.com/mrct) and the outcomes reported in the protocol will be compared to those reported in the final RCT publication. Industry-funded RCTs will be scored as ‘unclear’ for the other sources of bias criterion, due to the potential for funding bias [12].

### Synthesis of included studies

The data will first be summarized descriptively. If appropriate, pooled estimates of influenza illness will be derived using a random effects model [13] to calculate the relative risk. Vaccine efficacy will be calculated using the following formula: (1 - relative risk × 100). Separate meta-analyses will be conducted for the different types of influenza vaccine (that is, TIV, LAIV, other). Planned subgroup analyses include age group (children, adults, and older participants) and type of influenza (A versus B). Random effects meta-analyses will be conducted using SAS (SAS v9.2, SAS Institute, Cary, NC, USA) [13]. If extensive statistical heterogeneity is observed (for example, a statistically significant Cochran Q test for heterogeneity or an I^2^ statistic greater than 60% [12]) and the number of studies for outcomes of interest is greater than 10, we will conduct meta-regression analysis. The meta-regression analysis will explore the influence of factors such as age and baseline antibody levels on the meta-analysis results and will also be conducted in SAS [12].

## Discussion

This systematic review will comprehensively examine the extent to which influenza vaccines protect against non-matching circulating strains. Although previous influenza reviews exist [4-6,14], none have focused specifically on this issue. Furthermore, we will examine the degree of cross-protection separately for influenza A and influenza B, which has not been estimated previously. The results can be used by researchers and policy-makers to help predict the efficacy of influenza vaccines when they do not match circulating strains for a particular influenza season. Patients can use this information to decide whether to obtain the influenza vaccine and clinicians can use this information to decide whether to recommend the influenza vaccine for a particular season. This work will also provide valuable information to help inform the development of improved seasonal influenza vaccines.

This review is currently under way and there have been some changes to the protocol over time. The scope of the review, originally included crossprotection after vaccination and natural infection, however, after much deliberation, the team felt that this scope was too broad. Natural infection and vaccination are fundamentally different entities, with different types of studies examining each one. Therefore, it was decided to focus on vaccination only, thus narrowing the scope of the review. Furthermore, we initially planned to only include mismatch estimates of vaccine cross protection against influenza A and B. However, we discovered that little information is known about the matched vaccine efficacy estimates for influenza A and B separately. This information is not reported in previous influenza vaccine efficacy reviews, which only report aggregate vaccine efficacy against influenza A and B [4-6,14]. By conducting this additional analysis, we can clearly compare matched versus mismatched vaccine efficacy against influenza A and influenza B separately. Such distinctions will be important since it will be possible to explore the antigenic heterogeneity that exists within each subtype of influenza and the relevant impact on vaccine development.

Data on mismatch is not normally available in the title and abstract, therefore, all influenza trials were passed onto level 2 screening. As such, it was easy for the team to quickly go through the studies that were excluded at level 2 to identify the respective RCTs conducted during influenza seasons with matching circulating strains and vaccine composition.

The original plan was to conduct a systematic review on all years but it was felt that using the three Cochrane reviews on influenza vaccine efficacy among adults [4], older participants [5], and children [6] as a starting point would be a more efficient approach. The inclusion and exclusion criteria of the Cochrane reviews were consistent with the proposed review so it was felt that there was no sense in going back further in time. The team decided to obtain all of the included and excluded articles in the Cochrane reviews to make sure that potentially relevant material was not missed. Furthermore, all data obtained from the studies included in Cochrane were abstracted in duplicate by two reviewers to ensure accuracy (that is, we did not rely on the estimates published in the Cochrane reviews).

The literature searches were conducted from March 2006 (date of the most outdated Cochrane review search) until August 2011. They will be updated to February 1, 2012, to ensure all potentially relevant material is captured. The updated literature search will be conducted by Laure Perrier, an information specialist working with St. Michael’s Hospital, using the peer-reviewed literature search originally compiled by Dr. Jessie McGowan.

After reviewing another systematic review examining influenza vaccine effectiveness [15] the team came up with a sensitivity analysis for meta-analysis. This included the classification of influenza B mismatch into two different types: (1) mismatch due to within-lineage drift and (2) complete lineage mismatch (that is, B/Yamagata versus B/Victoria lineages) between the vaccine and the circulating strains. This will help explore antigenic heterogeneity related to influenza B and its impact on vaccine efficacy when using a mismatched vaccine, important information for vaccine development.

## Abbreviations

RCT: Randomized controlled trials; RR: Relative risk.

## Competing interest

This systematic review is funded by GlaxoSmithKline, Canada. ACT, DH, MT, CB, and ML have received consulting fees from GlaxoSmithKline. AC and GM are paid employees of GlaxoSmithKline

## Authors’ contributions

ACT, AC, GM, CB, and ML contributed to the conception and design of the review. ACT, DH, MT, CS will be involved in data acquisition of data. ACT and DH will analyze the data. ACT, AC, and ML, will interpret the results. All authors were involved in the drafting of this protocol and have given their approval for publication.

## Supplementary Material

Additional file 1 ** Appendix 1.** Draft eligibility criteria. Click here for file

Additional file 2 ** Appendix 2.** Draft medline search strategy. Click here for file

## Author details

^1^Li Ka Shing Knowledge Institute of St Michael’s Hospital, Toronto, Ontario, Canada. ^2^GlaxoSmithKline, Mississauga, Ontario, Canada. ^3^Faculty of Pharmacy, University of Toronto, Toronto, Ontario, Canada. ^4^Institute of Medical Sciences, University of Toronto, Toronto, Ontario, Canada. ^5^Applied Health Research Centre, St. Michael's Hospital, Toronto, Ontario, Canada.^6^North America Vaccines Division, GlaxoSmithKline, Philadelphia, PA, USA.^7^Department of Mathematics and Statistics, University of Guelph, Guelph, Ontario, Canada. ^8^McMaster University, Rm. 3203, 1200 Main Street West, Hamilton, Ontario, Canada.
