# Supplementary material for: Effect of influenza vaccines against mismatched strains: a systematic review protocol
Source: Syst Rev. 2012 Jul 30;1:35. doi: 10.1186/2046-4053-1-35 (PMC3488466; doi:10.1186/2046-4053-1-35)
Supplement: Additional file 1 — Appendix 1. Draft eligibility criteria. [file 2046-4053-1-35-S1.pdf]

APPENDIX 1: DRAFT ELIGIBILITY CRITERIA

Level 1 screening:

1. Is the study written in English?
   YES _____
   NO _____
   UNCLEAR _____

2. Are all participants healthy humans?
   YES _____
   NO _____
   UNCLEAR _____

3. Did the participants receive an influenza vaccine?
   YES _____
   NO _____
   UNCLEAR _____

4. Is this a randomized (or quasi-) randomized controlled trial?
   YES _____
   NO _____
   UNCLEAR _____

5. Is one of the treatment groups a placebo group?
   YES _____
   NO _____
   UNCLEAR _____

Level 2 screening:

1. Is the study written in English?
   YES _____
   NO _____
   UNCLEAR _____

2. Are all participants healthy humans?
   YES _____
   NO _____
   UNCLEAR _____

3. Did the participants receive an influenza vaccine?
   YES _____
   NO _____

UNCLEAR _____

4. Is this a randomized (or quasi-) randomized controlled trial?
   YES _____
   NO _____
   UNCLEAR _____

5. Is one of the treatment groups a placebo group?
   YES _____
   NO _____
   UNCLEAR _____

6. Is this a mismatched trial (i.e., the circulating strains do not match the vaccine composition)?
   YES _____
   NO _____
   UNCLEAR _____

Note: If you answer NO to any of these questions, the citation/study will be excluded. All other citations/studies will be included. We will keep track of reviews that have potentially relevant material and will scan their reference lists to ensure all studies have been captured.
