# Supplementary material for: Effect of influenza vaccines against mismatched strains: a systematic review protocol
Source: Syst Rev. 2012 Jul 30;1:35. doi: 10.1186/2046-4053-1-35 (PMC3488466; doi:10.1186/2046-4053-1-35)
Supplement: Additional file 2 — Appendix 2. Draft medline search strategy. [file 2046-4053-1-35-S2.pdf]

APPENDIX 2: DRAFT MEDLINE SEARCH STRATEGY

Database: Ovid MEDLINE(R) In-Process & Other Non-Indexed Citations and Ovid MEDLINE(R) <1948 to Present>:

----

1 Influenza Vaccines/

2 Influenza, Human/ or expInfluenzavirus A/ or expInfluenzavirus B/

3 exp vaccines/

4 2 and 3

5 ((influenza or flu) adj (vaccin$ or immuni$ or inoculat$ or shot)).tw.

6 1 or 4 or 5

7 randomized controlled trial.pt.

8 random$.tw.

9 7 or 8

10 6 and 9

11 limit 10 to english

12 animal/

13 human/

14 12 not (12 and 13)

15 11 not 14

16 6 and 7
